# Funder priority for vaccines: Implications of a weak Lockean claim

**DOI:** 10.1111/bioe.13075

**Published:** 2022-08-19

**Authors:** Anantharaman Muralidharan, G. Owen Schaefer, Tess Johnson, Julian Savulescu

**Affiliations:** ^1^ Centre for Biomedical Ethics Yong Loo Lin School of Medicine, National University of Singapore Singapore Singapore; ^2^ Oxford Uehiro Centre for Practical Ethics University of Oxford Oxford UK

**Keywords:** Lockean principle, overlapping consensus, vaccine distribution

## Abstract

The development of some COVID‐19 vaccines by private companies like Moderna and Sanofi‐GSK has been substantially funded by various governments. While the Sanofi CEO has previously suggested that countries that fund this development ought to be given some priority, this suggestion has not been taken seriously in the literature. Considerations of nationalism, sustainability, need, and equitability have been more extensively discussed with respect to whether and how much a country is entitled to advance purchase orders of the vaccine under conditions of absolute scarcity. Yet, little attention has been paid to whether prior investment into developing a vaccine entitles a country to some priority with respect to these orders. Moreover, while not a majority view, some survey results show that a significant minority of the populace does endorse some view like this. This article argues that the minority have a point: recognizing funder countries some priority is justified by the weak Lockean claim (WLC). According to the WLC, the fact that someone has contributed to the development of something gives them some entitlement to the resultant product. This article will defend the WLC, and address objections to the argument, including those pertaining to questions of historical injustice and medical need. This argument does not imply an unconstrained entitlement. Rather, contribution to development is one morally relevant factor that must be tempered by and weighed against potentially more substantial claims to priority based on need, equity, and other considerations.

## 1 INTRODUCTION

The COVID‐19 pandemic has raised urgent ethical questions surrounding the appropriate distribution of vaccines in conditions of scarcity, not only within each country's population, but also between countries. Some argue that vaccines should be distributed according to need, population size, or simply on a first‐come‐first‐served basis.^1^ However, there may also be justification for making vaccines available by priority to those countries that have a hand in their production. Here, then is a question of applied distributive justice: Does the fact that a country produced a vaccine give it some increased entitlement to those vaccines? To make this more precise, by “produce a vaccine,” we take the central cases to be ones where the state funded research and infrastructure for manufacture of the vaccine from tax dollars. It may be possible to extend the argument in this article to cover in‐kind contributions like participation in clinical trials and data‐sharing, but we refrain from doing so in this article owing to space constraints and the additional complexity such a discussion would involve.^2^ It may also be that it is in a given country's interests for the vaccine to be distributed to lower income countries instead. There is a discussion to be had about what countries should do with the vaccines that they are entitled to. However, in this article, we are only concerned to show that funding vaccine production generates some degree of entitlement. What a country should do with the vaccines it is entitled to is beyond the scope of this article. We also distinguish the financial contributions we are interested in from funding the manufacture of a vaccine by making advance purchase agreements. By increased entitlement, we mean that the state has a greater claim to accessing the vaccine than they would otherwise have had if access were merely based on that country's need, equity, nationalism or other morally relevant factors.^3^ This article attempts to answer this question in the affirmative.

Here is the strategy for this article. In Section 2, we discuss relevant background, and motivate the following discussion. Section 3 defends the weak Lockean claim (WLC) according to which, roughly, producers have increased entitlement to what they produce. Here we argue that this principle is entailed by most plausible moral frameworks by showing how it can be justified on consequentialist, autonomy‐based, and dignitarian grounds. Section 4 addresses three objections, namely, the historical injustice objection, the irrelevance objection, and the no opportunity objection.

## 2 BACKGROUND

The funding for vaccines is provided by states to a substantial degree.^4^ For instance, 37% of the development of the AstraZeneca COVID‐19 vaccine was directly funded by the United Kingdom while 34% was funded by other states.^5^ Development of the Pfizer‐BioNTech COVID‐19 vaccine received funding from Germany, but not the United States, as Germany funded BioNTech but the United States did not fund Pfizer. Meanwhile, the Moderna COVID‐19 vaccine's development was primarily funded by the United States, but not other countries. One complication is that while research and process development may occur in one country, the production of the vaccine may occur in another, and the packaging in yet another. Moderna, for instance, is manufactured in the Netherlands but put into vials in Spain. Astra‐Zeneca's vaccine is manufactured in India, the Netherlands and the United Kingdom. How should these contributions to the development and production of the vaccine affect its distribution?

One proposal is that countries that contribute to vaccine development and production are entitled to more of it compared to similarly placed countries that do not. On May 13, 2020, the CEO of Sanofi, Paul Hudson, indicated that the United States would receive priority access to the Sanofi‐GlaxoSmithKline vaccines if successfully developed, because the country had invested heavily in the development of the vaccine. Hudson later renounced this claim in response to pressure from the government and public of France, where Sanofi is headquartered.^6^ The backlash to Hudson's proposal seems to suggest that the claim that vaccine developers are at least *pro‐tanto* entitled to the vaccine is more controversial than it ought to be. While the considerations about who produced a vaccine have been given some weight by the public,^7^ it has received little serious attention in academic discussions of how vaccines are to be distributed.

Jecker and Atuire^8^ note that since the public has invested heavily in the science and upstream processes of translational research conducted by for‐profit companies, private companies are not the sole owners of the vaccine. Other discussions tend to focus on nationalist^9^ versus cosmopolitan views.^10^ In addition, while the import of other forms of contribution, for instance, by participating in trials or by providing virus samples is discussed in the literature^11^ no similar discussion has been had about monetary contributions.

This article discusses whether financial contributions like investments in research or manufacturing infrastructure entitle countries to some degree of priority in accessing a vaccine. Such priorities can come in a number of forms. For instance, countries could be entitled to extra advance purchase agreements, special discounts on purchasing the drug or export restrictions. We are not concerned about which form the priority takes, only whether producer countries are entitled to more of the vaccine, *ceteris paribus* than nonproducer countries.

What we do want to bracket is whether simply making an advance purchase agreement entitles a country to what they purchase. While there is a sense in which these advance purchase agreements do fund research, it ought not be assumed that any given country is entitled to buy up all the vaccines simply because they can. Rather, the focus of this article is on whether, as per Sanofi CEO Hudson, prior investment generates some entitlement to extra advance purchase agreements or to discounts or to exemptions from export restrictions, and so forth. If producing something generates entitlement to it, simply buying up everything via advance purchase orders can fail to do justice to contributions from those who did not make an advance purchase order. The bracketing strategy allows us to focus on what this prior investment entitles us to.

## 3 THE WLC

The argument in favor of our position is roughly Lockean.^12^ Consider the following:

1. In general, if someone funded (or otherwise contributed to) the development or production of something, all else equal, they have some additional entitlement to the produced good.
2. Country A funded the development and production of a particular good, the vaccine.
3. Country A, all else equal, has additional some entitlement to the vaccine (from 1 and 2).

The key premise in this argument is the first premise, which we shall call the WLC. It is Lockean in that it only narrowly entitles people to the specific products they fund the development or production of. For instance, the United States, which invested in Moderna's vaccine, but not Pfizer‐BioNTech's, has additional entitlement to Moderna's but not to Pfizer's vaccine. Countries that invested in vaccine candidates that turned out to be less effective or that did not pan out are not additionally entitled to the more effective versions that they did not invest in. This matches the way in which Lockean principles intuitively give rise to entitlements. For instance, suppose two farmers, Alf and Betty, put in the same effort to work two different plots of land. However, Alf's land generates a yield while Betty's does not. The mere fact that Betty also worked her land does not entitle her to an equal share of what is produced from Alf's. Of course, considerations of need and equity might entitle her to some of Alf's product, but she would be entitled to this regardless of whether she worked her land. It may even also be the case that the fact that Betty worked the land but did not succeed still entitles her to more than some third person who did not work their land. Nevertheless, all else equal, Alf would normally be entitled to more of his product than Betty is. That is to say, the WLC justifies some degree of inequality in how what Alf produces is to be distributed.

The WLC is weak in the sense that it leaves open the extent to which agents are entitled to what they fund the production of. For instance, it is compatible with significant positive duties of aid. The argument only entails that funder countries are entitled to more of the vaccine than they otherwise would be if they had not funded. They need not be (and probably are not) entitled to all of the vaccine they fund, because it is highly likely that there will be countervailing morally relevant factors that reduce their entitlement—for example, reduced need in comparison to other countries. The degree of priority is indeed limited, and may be entirely exhausted during acquisition of initial doses. This is relevant for boosters. Because boosters are at present mostly the exact same compound as initial doses, producer priority may not extend to entitling funders to any further priority for boosters. However, the WLC would potentially generate new entitlements should countries make additional investments for the development of boosters tailored to new variants.

It is also weak in that it does not entail a robust property right. Entitlement, instead, entails little more than the right to exclude. Very simply, an agent has the right to exclude others from an object if and only if (a) there is some action that ends up depriving others of their access to the object and (b) she has the right to perform that action. The right to exclude does not mean that all actions that deprive others of access are permissible, only that at least one is.^13^ The principle remains silent about rights to use, disposal, trade, and so forth. Hence even if a country is entitled to some number of vaccines as a result of the WLC, it might not have the right to dispose of them as it pleases. Perhaps it may only be permitted to use it for its own populace, give it away to other countries that need it or sell it at a reasonable price. That is, a person's entitlement to something is a claim right: others have an obligation to (a) refrain from interfering with her access to the object and (b) provide her with access if she previously did not have access to it. Thus, we might say that according to the WLC, funding the development of a good gives someone a defeasible claim right to the good she has funded. How then might we defend the WLC?

There are two things we might note. First, the WLC is something that we are already inclined to accept ordinarily in the individual case. For instance, every country considers it acceptable for people to keep at least some part of what they earn.

We might also point to another rough global consensus on tax rates here: capital gains taxes are usually between 0% and 30% with a maximum of 42% in South Korea. This suggests that nearly all countries think that people are entitled to more than half of their profit from investment income. This last point is important: the role that states play in funding vaccine development and production facilities is analogous to the role played by investors in a start‐up. While they do not actively research or produce the good in question, they provide a significant investment of capital. If, in the ordinary case, we think that people have a claim right to some significant chunk of their returns on investment, then so do countries that have invested in the vaccine.

This last claim might be objected to on the grounds that states might have duties that individuals or private companies do not have. For instance, the state may have duties to ensure a just distribution that abrogates property rights arising from production. However, this seems implausible. After all, if this were true, at least in this instance, egalitarians would be claiming that private investors aiming at private benefit could have more right to reap rewards than states aiming at public benefit. If anything, egalitarians would be expected to reverse the order of entitlement.^14^

At this point, someone might say that this only shows that people happen to accept the WLC, not that they are right to. This brings us to the second thing we might say in favor of the principle: it is justified on a variety of different grounds. In the rest of this section we argue that the WLC can be justified on, broadly speaking, consequentialist, autonomy‐based, and dignitarian grounds.

### 3.1 Consequentialism

The argument here is, loosely, rule consequentialist. The thought is that a world in which the WLC is not widely accepted is worse than one in which it is. To be clear, this last claim is ecumenical between different accounts of how certain distributions of wealth contribute to the world's goodness. The only accounts of distributive justice that are incompatible with the WLC will tend to be radically egalitarian ones according to which it is always better for goods to be equally distributed even when this results in everyone having less^15^ or ones where extremely small gains to the worst off can justify enormous losses to everyone else.

To see why, consider, first, the levelling down objection. According to this objection, an account of justice is excessively egalitarian if it regards one distribution of resource, B, as better than another, A, simply because the resources are more equally distributed in B than in A even though nobody in B has more of the resource than they would in A. Such conceptions of justice are said to achieve equality by levelling down; making some people worse off without making anyone else better off. Naturally, such levelling down, particularly in the context of vaccine distribution is objectionable. To illustrate, a world in which each country has 100 doses, intuitively, seems worse than a world in which LMICs have 100 each but HICs have 101 doses each.

Any distributive principle^16^ that is sufficiently moderate to avoid the levelling down objection will be compatible with the WLC. There are two parts to the explanation as to why this must be the case. The first part involves noting that the WLC is weak and compatible with extensive redistribution. The principle only requires that investors be entitled to more of the product than similarly situated noninvestors. It does not specify how much more. Hence, so long as redistribution does not fully equalize entitlements, the WLC is not violated. The second part of this explanation involves noting that in order to avoid the levelling down objection, a principle cannot endorse pareto inefficient distributions. That is, there cannot exist some achievable alternative distribution whereby some countries do better than in the endorsed distribution and no country does worse. This means that if there was a way to increase the production of a good relative to a given distribution and redistribute accordingly such that no country is worse off and at least some are better off, the distribution is not pareto efficient. As we shall argue, it is unlikely that anyone who currently lacks access to the vaccine would have access in the absence of the WLC. Therefore, the WLC is a necessary ingredient of almost any account of justice that is not vulnerable to the levelling down objection.

In support of the claim that no country is likely to be disadvantaged by the WLC, suppose that funding the development of a good did not entitle an agent to more of that good. She would, in general, have very little incentive to fund development if doing so did not entitle her to more of the good than if she had not funded it.^17^ After all, why spend considerable time, effort, or money investing in a good if she cannot realistically hope to enjoy some significant portion of such goods. Better to save herself some effort (and money) as she would be allocated an equal share anyway! Schmidtz's^18^ illustration of the Jamestown colony is educational: in 1607, when the colony was established, their charter entitled everyone to an equal share of what was produced regardless of contribution. That year two‐thirds of settlers died of starvation and disease. Subsequent voyages brought in more settlers to replace those who died, but more than 85% of settlers were dead of starvation and disease 2 years later. This situation persisted till 1614 when Governor Thomas Dale assigned to each person some land allowing them to keep what they produced. At this point, productivity increased sevenfold and the starvation came to an end. This shows that the disincentives created by equal shares were so severe that they resulted in mass death by starvation. Most of the early settlers would rather risk starvation and did starve instead of working for little profit.

The Jamestown colony case shows that incentives to produce, even if present, are too small under such radically egalitarian systems. After all, under equal distribution, working the land would have created some amount of food. And surely everybody receiving some amount of food would have been good for the local economy at the time and hence good for each individual farmer. Yet, any such incentives were insufficient to motivate people to work their land to any significant degree. If anything, given the fate of the Jamestown colony in its first 3 years, the incentives to produce would have been stronger than in the COVID case. After all, even in an egalitarian distribution, working the land would have given them *some* additional amount of food. Although the death toll from COVID has been quite serious^19^ it is not nearly as bad, percentage‐wise as the fallout from mass starvation in Jamestown. The incentive to produce under perfectly egalitarian distribution would have been even less.

Notably, we are assuming that countries and the people who run them are not perfectly motivated. They are not so purely altruistic that they will invest at comparable levels without some significant incentive. This is a reasonable assumption because HICs have been significantly un‐altruistic and quickly bought up vaccines regardless of whether they contributed to its development.^20^ The disincentive effect of slowing down vaccine innovation due to lack of government investment outweighs any marginal inefficiency (in terms of lives saved per dose administered) from giving some priority to funder countries. In any case, it is unlikely that anyone who currently does not have access to a vaccine would have received access to one under egalitarian distribution because it is unlikely that there would have been a vaccine.

One objection to the above argument is that we are comparing worlds in which there are different total numbers of vaccines. The proposal, instead, is to compare worlds with the same total number of vaccines. After all, the vaccines have already been made and the production facilities are already set up. Then, or so the objection goes, equal distribution will have better consequences for the worst‐off countries than the WLC.

However, this objection is short‐sighted. In subsequent pandemics, the world without the WLC will have fewer vaccines to distribute equally. Thus, over the long term, the worst‐off countries would be worse off in the world without the WLC than in the world with the WLC.

Another objection to the above argument is that norms like the WLC are not needed since existing power structures are already sufficient to generate the required incentives. Existing power structures exemplified by current capitalist societies guarantee that there is an incentive to fund production. Hence, or so the objection goes, the WLC is not necessary to generate the incentives.

However, this objection is mistaken because the mere existence of power relations is not sufficient to generate the right incentives. To see why, suppose, for instance, that the current power relations remained the same, but there was a strong egalitarian cosmopolitan norm whereby any country that came up with the vaccine would have to distribute it equally, for example, by giving it all to some agency like COVAX. There would be little incentive for anyone to come up with the vaccine because coming up with a vaccine would not be an effective way of putting a stop to the disease (for one's own country). The only reason existing power relations generate the incentives that they do have is because some norm consistent with the WLC is widely accepted and complied with.

Another objection along similar lines is that profits from selling the vaccine are sufficient to generate incentives for pharmaceuticals to invest in vaccine development. Hence there could be sufficient incentives even if vaccines were distributed equally. Our reply here is similar. The WLC is required to justify pharmaceutical companies keeping their profits. In a world in which nobody complied with the WLC pharmaceutical companies would not be able to keep their profits and hence would not have sufficient incentive to invest in vaccine development. Moreover, any consequentialist argument for pharmaceutical companies keeping their profits is also an argument for contributors in general being entitled to what they contribute to and hence for the WLC. If the WLC is true, then developer countries are entitled some priority access to the vaccine.

In fact, it is consistent with the WLC that existing arrangements allow funder countries to take more of the vaccine than the principle entitles them to. Hence, even if there would be adequate incentives to fund production of vaccines if funder countries were entitled to less than they currently are taking, the argument would still succeed as long as they need *some* degree of priority relative to similarly situated nonfunder countries in order to generate the incentives.^21^

A variant of the above objection is that being entitled to more of the profit instead of the goods is sufficient to generate incentives for countries to invest in vaccine development. After all, private investors invested in the vaccine without being promised priority in accessing the vaccine. Hence, or so the objection goes, priority access to the product is not required for incentives.

However, this objection errs in supposing that the vaccine is, in this instance, morally different from profit. After all, both are products of the investment with profit being downstream of the product itself. It is unclear why investors are morally entitled to a greater share of profits but not a greater share of the goods they invest in. If, between profits and goods, investors were entitled to only one, it is more likely to be goods since the latter is a more direct result of the investment. They are entitled to the profit only derivatively of being entitled to sell their share of the good they are entitled to. If, for any reason, investors lacked the right to sell a given good, they would be entitled to only the goods but not the profit. Conventionally, investing yields a share of profits only because money is fungible, not because there is anything morally special about profits. Hence, it should be permissible for an investor country to forgo profit in favor of increased access to the vaccine.

This parity between vaccine and profit also reveals that contrary to the objection, being entitled to more of the profit is consistent with the WLC. After all, countries could sell the vaccine to financially recoup their investment. If they had received money instead, they could use that extra money to buy up more vaccines from any LMIC willing to sell them.^22^ As such, any incentive to invest in vaccines that private investors have is owed to arrangements consistent with the WLC. If the foregoing is right, then profit is equivalent to priority access to vaccine because it makes the vaccine more accessible. Therefore, priority access to vaccine, in some form or other, is necessary to generate the requisite incentives.

Even if profit was not equivalent to vaccines, when thinking of incentives, it is important to take into consideration what the relevant actors really want. In the current case, countries chose vaccines over profits for good reason. The economic and human cost of the pandemic far exceeded the profits that may have been gained from the vaccine. To get some handle on the numbers involved, the economic losses in 2020 alone were estimated at almost US$3 trillion.^23^ By contrast, the revenue (not profit!) from all COVID‐19 vaccines can be estimated at about US$54 billion.^24^ There are two orders of magnitude difference between the two amounts. For countries that are able to effectively distribute their vaccines, it makes sense that they would be incentivized to fund development more by access to vaccines than by a share of profits. Therefore, we can incentivize investment in vaccines by countries significantly better by according some priority access to vaccines than by some share in profit.

Notably, the argument from incentives does not require us to endorse consequentialism to succeed. Any view in which the value of the outcome is at least one of the things that matters will also accept the argument. In addition, any contractarian view will accept the argument as well. After all, the consequences caused by the disincentives are likely bad for everyone and hence no rational agent would reject the WLC.

Similarly, according to eudaimonic virtue theories, virtues are dispositions that are conducive to human flourishing. If people were disposed to distribute what they funded equally to funder and nonfunder alike, there would be little incentive to invest. Hence, such bad outcomes would mean that dispositions to act in ways contrary to the WLC are vices. Likewise virtuous behavior in distributing goods must involve dispositions to act that are consistent with the WLC.

### 3.2 Autonomy

The autonomy‐based justification is as follows: the degree to which an agent's activities are self‐directed is greater when she is entitled to the product of her own investment than when she is entitled to the product of another's investment instead. Autonomy, broadly understood, is about setting the direction of one's life to some degree. According to Raz “The ideal of personal autonomy is the vision of people controlling, to some degree, their own destiny, fashioning it through successive decisions throughout their lives.”^25^ We have greater autonomy if the direction our lives takes is, to a greater degree, a product of the choices we make.

To illustrate the above point, suppose that we were instead not entitled to what we invested in, but instead entitled to what someone else invested in. If that were so, then if a person wanted to use a particular tool, she would have to depend on whether someone else chooses to invest in the production of the tool. Even if, for instance, Alf was entitled to what Betty invests and, luckily, Betty happens to invest in the tool, Alf's obtaining the tool and using it is less self‐directed than in the case where he funds the production of the tool himself. In addition, if Alf would receive the tool regardless of whether he invested in any, then his life outcomes are not a product of his decisions. Hence, if we care about the extent to which our activities are self‐directed, then people ought to have a claim right to exclude others from some of what they invest in.

The above argument can be readily extended to cover states as well. On one approach, we might suppose that the state's autonomy interest is a proxy of the autonomy interests of the citizens who comprise the country and whom the state represents. We will assume here that state decisions to invest in the vaccine and receive priority access enjoy a sufficient degree of democratic legitimacy. In such cases, state decisions to fund a vaccine are not incidental to citizen decisions. If, as a result of their own decisions, citizens gain access to a vaccine, their lives are more self‐directed than if they had to depend on the decisions and preferences of the citizens of other countries. Where the citizens opt for an egalitarian distribution instead, it might, for reasons of legitimacy, be wrong for states to negotiate priority access. However, this would not be because the WLC is false, but instead only because these sorts of preferences may render contrary policies illegitimate. People are, after all, free to waive their rights to things they are entitled to. It does not change whether states are permitted to negotiate some degree of priority access provided that doing so would be acceptable to its citizens.

On another approach, we might loosen our austere individualist constraints and suppose that all agents, whether individuals or group agents like states, have autonomy interests. An interest in autonomy arises from an agent's interest in her life outcomes tracking her decisions in some way. However, having an interest in one's life outcomes tracking one's decisions is constitutive of being an agent. There is no point in being an agent if one's decisions had no bearing on how one's life turned out. If this is right, then the fact that an agent is a group and not an individual does not detract from its autonomy interests.

One might object that since group agents like states and corporations do not have lives as such, they cannot have interests in life outcomes tracking their decisions. Yet, this would be too quick. Group agents typically have charters or constitutions that specify certain overarching goals. The goals would be the things the group agent exists to achieve and it would exercise its agency throughout the period in which it is constituted to achieve them. These goals are analogous to the life goals that individual agents have and that they make decisions in order to achieve. If, as a result of these life goals, individual agents have an interest in the extent to which their achievement of these goals tracks their decisions, then there is no reason to think that group agents that have analogous goals do not also have an interest in the extent to which their achievement of these goals tracks their decisions.

At this point, one might object that even if this is true, the autonomy of people in low‐income countries could be significantly enhanced by distributing the vaccines equally. After all, this would allow them to live their lives according to their life plans instead of falling ill and dying. Thus, or so the objection goes, one could get more self‐direction in the aggregate by violating the WLC than by abiding by it.

This objection, however, ends up treating autonomy as a value to be promoted instead of respected. Whereas promoting autonomy is compatible with trading off the autonomy of one person's actions against the autonomy of other actions taken by that person and others, this is not the case with respecting autonomy. For instance, if autonomy ought to be promoted, we may override a person's decision to not take mind enhancing treatments in order to enhance their capacity to give informed consent in other circumstances. Respecting autonomy means not overriding consent in such circumstances. However, the claim that autonomy ought to be promoted is a controversial assumption. It is more plausible that autonomy ought to be respected, not promoted. Violating the WLC is inconsistent with respecting autonomy because the autonomy of the funder country's decision to invest is traded off against the autonomy of people in low‐income countries.

Even if autonomy ought to be promoted, the objection does not succeed. After all, the only way in which autonomy could be promoted is if fewer people fall severely ill by violating the WLC. However, this is unlikely to be the case. As we have argued earlier, violating the WLC is unlikely to result in more vaccines for low‐income countries because there would be too little incentive to invest in vaccines.

We need not be Raz‐ians in order to accept the argument from autonomy. On the Rawlsian picture, we have fundamental interests in exercising and developing our two moral powers. The crucial power for this argument is the first one: the capacity to form, revise, and pursue one's conception of the good. In the Original Position, the principles of justice are chosen from behind a veil of ignorance by rational agents who aim at pursuing their fundamental interests. Exercising and developing the first moral power involves agents choosing projects, pursuing them, and revising their goals in light of the conflicts with their own other projects and others'.^26^ However, an agent's capacity to pursue her conception of the good would be severely curtailed if she was not entitled to what she produced or invested in. Hence, a greater right to the products of her contribution would be among the rights included in any plausible Liberty principle agreed to in the Original Position.

One objection to the above claim is that the WLC would not be chosen if the parties knew that they would be inhabiting a nonfunder country. There are three things we can say in response. First, in the Original Position, the parties behind the veil of ignorance do not know specific information about the society they live in,^27^ only general facts in the social sciences. Such specific information is only revealed at a later stage of justification.

One might reply that they know that they *might* end up in a nonfunder country and hence apply the Difference principle, which would in turn require a more egalitarian distribution. This brings us to the second reply, namely, that given that the WLC has been incorporated within the Liberty principle, it enjoys a strict priority over the Difference principle. The reason for this is that the Liberty principle protects rights that are directly necessary for the exercise and development of the two moral powers. This means that even *if* the Difference principle would require more redistribution of vaccines, the Liberty principle makes such redistribution impermissible. Moreover, as already argued, suitably understood, there is no real conflict between the WLC and the Difference principle. Coupled with an adequate amount of redistribution, the incentives generated by giving funders some degree of priority will be to the benefit of the worst‐off countries.

Third, the WLC governs many more cases than just the distribution of vaccines. Even if the parties knew in advance that they would end up in a nonfunder country, without the WLC, they would lose out on many opportunities to form, revise, and pursue their conception of the good.

In addition, on the Scanlonian picture, the rule that we should not give funders priority, arguably, can be reasonably rejected. Funders have a substantive interest in keeping what they invest in. A plausible way to cash out this interest is by relating it to autonomy. While everyone, funder and nonfunder alike has some prudential interest in some good, only funders have an additional autonomy‐related interest in keeping what they invest in. While it may not be reasonable for them to insist on keeping everything, it seems equally or even more unreasonable to expect them not to enjoy any priority in accessing the goods they invest in.

In short, there is a constellation of views in which autonomy plays an important role. On any such view, the WLC would be plausible. Let us now turn to a third route by which we could justify the WLC.

### 3.3 Dignity

Dignitarian accounts focus on agents’ moral status. The argument here will be given a Kantian gloss, but non‐Kantian accounts should serve as well. People make things in pursuit of some particular end. If we reject the WLC, we make the claim that funders of a product are not any more entitled to the product than anyone else. We thereby demand of them that they give up most of what they have funded and disregard their own reasons for investing in the good. We thus treat them (or more precisely, their rational natures) as mere sources of the good and not ends in themselves. Similarly, when an agent regards herself as obligated to not give herself priority access for what she invests in, then she does not take herself seriously as a being with aims and reasons to act. Instead, she ends up regarding herself as a mere source of funds/goods, a cypher interchangeable with others.

Here is an objection to the above argument: Is it not the case that funders are treated merely as a means only if they did not have any access at all to what they produce? Since they invest in the good in order to do something with it, as long as they have some entitlement to the goods they fund, are they not also treated as an end?

Our reply here is that there is a significant difference between funders and nonfunders: the former exercised their capacity to respond to reasons in investing in the good. In supposing that their entitlement does not exceed nonfunders’ we are, on the one hand, taking advantage of the fact that production and development of the good have been funded without acknowledging the fact the funders exercised their rational capacities in a way that resulted in the good. The only way we could justify equal entitlement for funders and nonfunders alike is if we regarded the exercise of the capacity to reason as an irrelevant fact. After all, if it was a relevant fact, then it should make a difference to what they are entitled to. Hence, if, by hypothesis, it does not make a difference, it must not be relevant. Yet, to treat a person's exercise of this capacity as irrelevant is, necessarily, to fail to treat that person's rational nature as an end in itself.

A related objection is that being entitled to a share of the profits instead of the vaccine would involve treating funders as ends in themselves while rejecting the WLC. However, as already discussed earlier, this arbitrarily distinguishes between the product and the profit from selling it. There is no reason to think that funders are entitled to the latter without being entitled to the former.

If the arguments we have presented so far succeed, then we can say that the WLC is the subject of an overlapping consensus of reasonable views. Most reasonable moral views will invoke some broadly consequentialist, autonomy‐based, or dignitarian considerations, or perhaps even more than one such consideration. If this is right, it would be unreasonable to reject the WLC. Given the WLC, if a given country invested in the development or production of a vaccine, it would be more entitled to the vaccine than a similarly situated country that did not invest in said vaccine.

## 4 MORE GENERAL OBJECTIONS TO THE ARGUMENT

Any objections that Section 3 dealt with pertained to specific theories/arguments that might be used to justify the WLC. This section will deal with more general objections to the argument spelled out in Section 3. The three objections we will discuss are the historical injustice objection, the irrelevance objection and the no opportunity objection. We shall present and respond to each objection in turn.

### 4.1 Objection 1: Historical injustice

One objection is that prioritizing wealthy countries that can afford to fund vaccine development entrenches global injustice. More specifically, the existence of historical and ongoing injustices undercuts Lockean property claims. The legitimacy of Lockean claims depends on there being a more or less unbroken link of just transfers between just original acquisition, and current title. However, many rich countries acquired their wealth by exploiting other countries. This breaks the link and hence they are not entitled to this wealth nor are they entitled to the proceeds from investing this wealth.

There are five ways in which we might reply to this objection. First, we might say that whilst historical injustices may figure into determining entitlement as well, this does not *negate* additional entitlement by virtue of being a funder, but rather merely counts as a negative in the equation of certain funders’ overall entitlement.

In other words, the objection proves too much. If the mere existence of some historical or ongoing injustice in the history of some good completely undermines Lockean entitlements to the good, then we would not be able to justify any form of property. The reason for this is that any form of property inconsistent with the WLC is ruled out by consequentialist, autonomy‐based, and dignitarian considerations. This would commit us to nationalizing big pharmaceutical companies or abolishing private property entirely. Few are willing to bite this bullet.

Somewhat more controversially, the current system of private property and production of pharmaceuticals is at least partly responsible for the record time in which the vaccine was developed. If there had just been one single global effort at searching for the vaccine instead of the multiple attempts, there is a good chance that the vaccine would still have not been ready at the point this is being written. After all, of the many different attempts at developing a vaccine, only a few were successful. If this is right, then historical (and maybe even ongoing) injustices are not such that they completely undermine *all* property claims. Hence, while the degree of entitlement may be reduced, it is not completely negated. Thus, the fact of investment in production and development still generates *some* degree of entitlement. Exactly how much this is, would be the exercise of another paper.

Second, we might argue that even if the legitimacy of the full bundle of property rights is undermined, it does not follow that no element of that bundle remains. It's entirely possible that this particular element, the right to exclude people from the physical product, might remain.

Third, we might think that any plausible account Lockean entitlements includes a “statute” of limitations. As the wrongdoing recedes further into the past, the degree to which that wrongdoing undercuts entitlement claims decreases. This is especially true where there are multiple wrongdoings in the chain of transfer of entitlements. Suppose, for instance, that some piece of land was initially obtained unjustly (e.g., by violent means), then, it was subsequently acquired by another country, also by violent means and so on. Most cases are like this wherein no one has clean hands. In such cases, the original rightful owner is lost to history. The logic of the Lockean account is such that since we cannot properly assign rightful ownership to anyone, it belongs to no one and hence returns to the commons. If at some point it returns to the commons, then some of the transformation of raw materials to useable products is a kind of legitimate original appropriation. As such, by the WLC, legitimate transformation of unowned resources by labor or investment of one's own legitimate property generates at least some degree of entitlement to the produced good.

Fourth, the objection seems to implausibly imply that we are never responsible to any degree for our current circumstances. It is implausible that previous historical conditions of exploitation/oppression, and so forth are so causally efficacious that they continue to immiserate a country well after that oppression and exploitation have been ameliorated. While some of the current poverty in the global south might be a legacy of previous historical injustice inflicted by the global north, not all of it is. Rather, at least some of their current conditions are the result of local and regional neglect, corruption, oppression and exploitation. Given that HICs are not responsible for the local and regional wrongs perpetrated on LMICs (or at least not for all of it), their Lockean entitlement is not reduced by those injustices. Acemoglu and Robinson estimate that one third of global inequality is attributable to colonialism.^28^ If inequality caused by colonialism can be disentangled from other causes of inequality, then we can also further disentangle other sources of historical injustice. If this is right, then there is still some substantial degree to which we are entitled to what we invest in.

The egalitarian might object that we can in fact point to more recent injustices that have caused countries, especially those in Africa, to be unable to fund vaccine development. Jecker specifies three instances of such injustices. First, concessionary lending practices by the World Bank and IMF gutted health and educational infrastructure because recipients were forced to cut spending on health and education in order to repay the loans on time. Second, the agreement on trade‐related aspects of intellectual property rights (TRIPS) intellectual property framework sharply curtailed the ability of poorer nations to access innovations in healthcare. Third, even well‐intentioned measures like COVAX ended up displacing effective enforcement mechanisms for distributing vaccines.^29^

This brings us to the fifth response we might give: the right response to historical or ongoing injustices need not involve violating the WLC. As Jecker herself notes,^30^ the appropriate restitution for these injustices does not itself involve redistributing vaccines from HICs to LMICs.

The remedy for concessionary loans involves setting standards for loans that develop health infrastructure. Indeed, with falling vaccine demand from HICs, one of the biggest bottlenecks to vaccinating countries in Africa is the lack of ability to store, transport, and administer vaccines.^31^ This is consistent with the WLC as improving infrastructure in LMICs is consistent with developer countries getting some degree of priority access.

The remedy for TRIPS is to waive intellectual property rights^32^ for vaccines during the pandemic. This too is consistent with the WLC. After all, the principle is about being entitled to the physical objects that one has contributed to the development of, not the right to exclude others from using those ideas and duplicating one's products. Hence Germany, which funded BioNtech's research should get some priority in accessing the product of this research while Pfizer and BioNtech^33^ is obliged to offer restitution by declining to enforce its patent. Both conditions can be jointly satisfied. Pfizer and BioNtech can give Germany a discount or set aside some additional share of the doses it produces to them. At the same time, it can make its formula for the vaccine public. Doing one need not preclude the other.

The remedy for COVAX's displacement of enforcement mechanisms is to establish effective enforcement mechanisms for vaccine distribution. The WLC is consistent with this because the principle is about which norms ought to be enforced. In fact, the WHO has a better chance of getting buy‐in for an enforceable arrangement from all of its member states if there is some degree of developer priority than if there is not.

To sum up, all of Jecker's proposed remedies to recent or ongoing injustices are consistent with having some degree of priority for developer countries. The injustice that developer countries inflict on LMICs would have to be significantly greater, more direct, and specific to the vaccine in order for their Lockean claim to be completely negated.

### 4.2 Objection 2: Irrelevance to the COVID‐19 situation

The second objection here is that while the WLC is a perfectly acceptable principle for our everyday interpersonal interactions and even for global principles of distributive justice, it is irrelevant for our current COVID‐19 situation. Instead the current situation is one of extreme urgency, more akin to the situation in an emergency where patients are triaged according to medical need, not ability to pay or whether they produce medicines for treatment or like war where property is appropriated and resources are used to do the most good. In such situations, or so the argument goes, the only consideration is what will be most effective at resolving the emergency.

The reply to this objection has two parts. First, there is an important difference between triage situations and our current situation: while the current need for vaccines is rather urgent (especially in the global south), triage situations are genuinely zero‐sum. How we choose to distribute medical resources in such situations does not create bad incentives and affect future availability of medical supplies, nor, for that matter, does it implicate the autonomy, or dignity of the producers of medical supplies. That is to say, whereas in triage situations, the producer or funder is not directly in the equation, this is not true in our current COVID‐19 vaccine distribution situation. By contrast the question of how we distribute access to vaccines does interact with these other considerations.

While this reply works with the triage comparison, it does not work as well for the war comparison where, historically, property has often tended to be appropriated from producers and investors.^34^ Here, a different argument will be provided. The first thing to note is that while there is a long history of expropriation during times of war, it is far from clear that such actions are permissible, rather than merely expedient. Historically, people have tended to be very willing to do terrible things in times of war. The mere fact that we are willing to expropriate property during war time does not mean that it is actually permissible to do so in those circumstances, let alone ours.

This is not to say that it is never permissible to appropriate property in war times. We can clearly imagine *some* such situations. However, the circumstances in which this would be permissible are sharply circumscribed. For instance, the war must be just, the appropriation must be genuinely necessary to prevent some terrible consequence or injustice, and doing so will be effective. Specifically, in war time, the WLC can be violated only to stave off significantly worse consequences, severe reductions in autonomy or violations of dignity. While the COVID‐19 situation is serious, it is hardly so dire that it would fit the stringent conditions that permit us to violate the WLC.

Even if considerations of efficacy dominate to the exclusion of all other considerations, this does not mean that the WLC can be violated. The key reason is that one of the grounds upon which the WLC is justified is the goodness of its consequences. Abiding by the WLC is probably the most effective way of hastening vaccine discovery and maximizing vaccine production. As long as vaccine discovery and production are slow, the situation will persist.

### 4.3 Objection 3: No opportunity to produce

One might object that the WLC might justify some degree of priority to funders or producers, but only if others had an opportunity to fund or produce the good. However, it may seem unfair to deny an equivalent WLC for LMICs that would have substantially invested in vaccine development if feasible, but they simply could not reasonably afford such investment.

In reply, there are two things we might say here. First, this objection leaves in place a WLC between countries that had opportunities to fund vaccine development. Indeed, many medium and high income countries had adequate opportunities to fund vaccine development but did not. For instance, the United States did not directly fund the development of the Pfizer‐BioNtech vaccine.^35^ It would be unfair for the United States to have the same level of access to the Pfizer‐BioNtech vaccine as Germany which did fund the vaccine just because much poorer countries did not have the opportunity to fund the vaccine.

Second, none of the three justifications that we have provided for the WLC are affected by some people not having the opportunity or resources to invest in a good. Even if some people lack opportunities to invest, some degree of priority is still needed to generate adequate incentives for those that do have opportunities but may decide not to exercise them.

Consider now, the autonomy based justification. While people who lack the opportunity to invest lack one way in which to control their lives, denying the WLC would not give them these opportunities. Moreover, the WLC is compatible with redistributive policies that would give people the opportunity to invest. Most importantly, just because one party lacks the opportunity to produce or invest, it does not follow that giving another party some degree of priority does not respect his autonomy. After all, without the appropriate degree of priority funders’ outcomes would not reflect their choices. The existence of those who lack the opportunity does not negate the fact that WLC ensures a degree of autonomy for those who do have the opportunity.

Consider, lastly, the dignitarian justification. The mere fact that some people lacked the opportunity to invest does not license treating funders merely as means to an end by not giving them any priority access to the good they produce.

To sum up, if any of the discussed justifications are successful when everyone has the opportunity to invest in production/development, they still work when only some people have that opportunity. Redistributive measures might be needed to ensure that everyone has adequate opportunities, but this need not be inconsistent with the WLC. Additionally, the WLC is needed to explain why it would be unfair for rich nonfunder countries to be entitled to the same amount as rich funder countries.

## 5 CONCLUSION

Summing up, we have provided a number of arguments to suggest that the WLC is true. Crucially, any plausible moral theory entails the WLC. We have also presented some objections to the above arguments and argued that these objections do not succeed. If all the arguments above succeed, countries that invest in vaccine development get some degree of priority access to the vaccines.

To place things into context, considerations of need, alleviating suffering, and so forth are not categorically overridden by the WLC. Rather, the fact that a country has funded the development or production of a vaccine should count for something. Exactly how much this counts for will depend on what the correct justification for the WLC is. Nevertheless, whichever justification is correct, there will be some sizeable degree to which funder countries are additionally entitled to access the vaccines they produce. After all, without a sizeable entitlement, the incentive to invest may not be adequate, autonomy may be infringed significantly and people may not be sufficiently respected as ends in themselves. At the very least, we can say that nonproducer countries that are not in dire need have less entitlement to access the vaccine than funder countries.

## CONFLICT OF INTEREST

Julian Savulescu is a Partner Investigator on an Australian Research Council grant LP190100841 which involves industry partnership from Illumina. He does not personally receive any funds from Illumina. Julian Savulescu is an ethics consultant for Avon Cosmetics Ltd (2021‐2023). Julian Savulescu is a Bioethics Committee consultant for Bayer.

## Footnotes

1 See Williams, J., & Dawson, A. (2020). Prioritising access to pandemic influenza vaccine: A review of the ethics literature. *BMC Medical Ethics, 21*, 40. https://doi.org/10.1186/s12910-020-00477-3; Emanuel, E. J., Persad, G., Kern, A., Buchanan, A., Fabre, C., Halliday, D., Heath, J. Herzog, L., Leland, R. J., Lemango, E. T., Luna, F., McCoy, M. S., Norheim, O. F., Ottersen, T., Schaefer, G. O., Tan, K.‐C., Wellman, D. H., Wolff, J., & Richardson, H. S. (2020). An ethical framework for global vaccine allocation. *Science, 369*(6509), 1309–1312.

2 It may turn out that the Lockean principle we articulate and defend in this paper entails that countries like South Africa that serve as testing sites for a vaccine are entitled to some priority access to that vaccine.

3 Certainly, the fact that a country funded the vaccine development needs to be balanced against other considerations like need, equity, reducing global spread of disease, and so forth. This article only claims that this fact needs to be given some weight.

4 Kiszewski, A. E., Cleary, E. G., Jackson, M. J., & Ledley, F. D. (2021). NIH funding for vaccine readiness before the COVID‐19 pandemic. *Vaccine, 29*, 2458–2466.

5 Safi, M. (2021, April 15). Oxford/AstraZeneca Covid vaccine research was 97% publicly funded. *The Guardian*. https://amp.theguardian.com/science/2021/apr/15/oxfordastrazeneca-covid-vaccine-research-was-97-publicly-funded

6 Bostock, B. (2020, May 15). *Sanofi walked back a promise to give the US priority access to its coronavirus vaccine after outrage from the French government*. Business Insider. https://www.businessinsider.com/coronavirus-vaccine-sanofi-scraps-us-priority-france-outrage-macron-2020-5?r=US&IR=T

7 Klumpp, M., Monfared, I. G., & Vollmer, S. (2021). To share or not to share ‐ What the general public thinks about global COVID‐19 vaccine distribution. *MedRxiv*. https://doi.org/10.1101/2021.08.16.21262116

8 Jecker, N. S., & Atuire, C. (2021). What's yours is ours: Waiving intellectual property protections for COVID‐19 vaccines. *Journal of Medical Ethics, 47*(9), 595–598. https://doi.org/10.1136/medethics-2021-107555

9 The WLC has a wider scope in application than nationalist claims. An entitlement generated by the WLC creates duties for third parties to refrain from interfering with the funding country's access. This can only be weighed against claims that similarly apply to third parties, such as need or equity. Nationalistic prerogatives do not generate obligations for third parties to respect the entitlement. As such, the WLC may be relevant in contexts where nationalism is not applicable, such as when third parties are deciding how to allocate scarce supplies of vaccine between different countries.

10 Jecker, N. S., Wightman, A., & Diekema, D. (2021). Vaccine ethics: An ethical framework for global distribution of COVID‐19 vaccines. *Journal of Medical Ethics, 47*, 308–317.

11 Fidler, D. P. (2010). Negotiating equitable access to influenza vaccines: Global health diplomacy and the controversies surrounding avian influenza H5N1 and pandemic influenza H1N1. *PLOS Medicine, 7*(5), e1000247. https://doi.org/10.1371/journal.pmed.1000247

12 On Locke's account of property, one way in which we could legitimately come to acquire property rights in an object is by producing said object. See Nozick, R. (1974). *Anarchy, state and utopia*. Blackwell, for a more recent exposition. While Locke explicitly talked about labor, the justificatory structure of the arguments we produce for the claim obviously extend to investments as well.

13 By contrast, someone possesses the right to use, disposal, trade, and so forth only if they have the right to perform any (or almost any) action that counts as using, disposing of, trading, and so forth.

14 See Emanuel, E., Buchanan, A., Chan, S., Fabre, C., Halliday, D., Heath, J., Herzog, L., Leland, R. J., McCoy, M. S., Norheim, O. F., Saenz, C., Schaefer, G. O., Tan, K.‐C., Wellman, C. H., Wolff, J., & Persad, G. (2021). What are the obligations of pharmaceutical companies in a global health emergency? *Lancet, 398*, 1015–1020. https://doi.org/10.1016/S0140-6736(21)01378-7

15 Even so, most egalitarians are keen to avoid the levelling down objection.

16 For instance, consider the leximin principle according to which the prospects of the worst off are maximized, then holding that constant, the prospects of the second worst off, then the third and so on. See also Wagstaff, A. (2002). Inequality aversion, health inequalities and health achievement. *Journal of Health Economics, 21*(4), 627–641; Broome, J. (2002). Chapter 3.5: Fairness, goodness and levelling down. In D. Wikler (Ed.), *Goodness and fairness* (pp. 135–137). Oxford University Press, for hybrid measures of outcomes that combine utilitarian and egalitarian concerns.

17 Grafton, R. Q., Squires, D., & Fox, K. J. (2000). Private property and economic efficiency: A study of a common pool resource. *Journal of Law and Economics, 43*, 679–713.

18 Schmidtz, D. (1994). Institution of property. *Social Philosophy and Policy, 11*, 42–62.

19 Mortality rate for COVID‐19 at the time of writing stands at about 2%.

20 See Zhou, Y. R. (2022). Vaccine nationalism: Contested relationships between COVID‐19 and globalization. *Globalizations, 19*(3), 450–465; Callaway, E. (2020, August 27). The unequal scramble for coronavirus vaccines – By numbers. *Nature*. https://www.nature.com/articles/d41586-020-02450-x; Bhutto, F. (2021, April 4). The world's richest countries are hoarding vaccines. This is morally indefensible. *The Guardian*. https://www.theguardian.com/commentisfree/2021/mar/17/rich-countries-hoardingvaccines-us-eu-africa

21 A further question may be raised about whether pharmaceutical companies are retaining excessive profits compared to what is necessary to incentivize research and development, and as such whether certain intellectual property protections should be waived. See Jecker & Atuire, op. cit. note 8. However, such questions are outside the scope of our argument, which focuses on country rather than pharmaceutical company entitlement.

22 Given the high rates of vaccine and COVID skepticism in Democratic Republic of Congo and other places in the global south, we can easily imagine that more than a few countries would have been willing to take them up on that offer.

23 Szmigiera, M. (2022, May 26). Impact of the coronavirus pandemic on the global economy – Statistics & facts. *Statista*. https://www.statista.com/topics/6139/covid-19-impact-on-the-global-economy/

24 Richter, F. (2021, December 7). Despite Covid, vaccines account for minor share of pharma sales. *Statista*. https://www.statista.com/chart/26350/vaccine-sales-as-a-share-of-global-pharmaceutical-sales/

25 Raz, J. (1986). *The morality of freedom*. Oxford University Press, p. 369.

26 This matches the Raz‐ian gloss Tomasi gives to the first power as being about responsible self‐authorship in Tomasi, J. (2012). *Free market fairness*. Princeton University Press. See also Lomasky who makes a similar argument in Lomasky, L. E. (2005). Libertarianism at twin Harvard. *Social Philosophy and Policy, 22*(1), 178–199.

27 Rawls, J. (1999). *A theory of justice, revised edition*. Harvard University Press, p. 118.

28 Acemoglu, D., & Robinson, J. A. (2017). The economic impact of colonialism. In S. Michalopolous & E. Papaioannou (Eds.), *The long economic and political shadow of history* (pp. 81–87). CEPR Press.

29 Jecker, N. S. (2022). Global sharing of COVID‐19 vaccines: A duty of justice, not charity. *Developing World Bioethics*, 1–10.

30 Ibid: 9.

31 While earlier articles like Kiecol, K. (2021, October 27). *World Health Summit: Vaccination rate in African countries too low*. Malteser International, point to vaccine supply as the key limiting factor, more recent articles like Mwai, P. (2021, December 31). *COVID‐19 vaccinations: African nations miss WHO target*. BBC News; Arezki, R. (2022, January 14). *It is time for Africa to focus on getting vaccines in arms*. Al‐Jazeera; Milko, V. (2022, March 4). *Why are COVID vaccination rates still low in some countries?* Associated Press, emphasize infrastructure deficiencies as the key bottleneck in achieving vaccination goals.

32 See also Hettinger, E. C. (1989). Justifying intellectual property. *Philosophy and Public Affairs, 18*(1), 31–52 for a criticism of intellectual property rights.

33 Moderna has in fact promised not to enforce its patent for the COVID vaccine.

34 Marcus, P. (1942). Taking and destruction of property under a defense and war program. *Cornell Law Review, 27*(3), 317–346.

35 Griffin, R., Drew, A., & Bloomberg. (2020, November 10). Germany funded the development of Pfizer's COVID vaccine – Not U.S's Operation Warp Speed. *Fortune*. https://fortune.com/2020/11/09/pfizer-vaccine-funding-warp-speed-germany/

